# Dichloridobis(1,3-diisopropyl-4,5-di­methyl-1*H*-imidazol-3-ium-2-thiol­ate-κ*S*)copper(II)

**DOI:** 10.1107/S1600536813006879

**Published:** 2013-03-16

**Authors:** Ulrich Flörke, Aziza Ahmida, Jörg Schröder, Hans Egold, Gerald Henkel

**Affiliations:** a Department Chemie, Fakultät für Naturwissenschaften, Universität Paderborn, Warburgerstrasse 100, D-33098 Paderborn, Germany

## Abstract

The mol­ecular structure of the title compound, [CuCl_2_(C_11_H_20_N_2_S)_2_], shows the Cu^II^ atom with a distorted tetra­hedral geometry from two Cl atoms [Cu—Cl = 2.2182 (6) Å] and two thione S atoms [Cu—S = 2.3199 (6) Å]. The angles at the copper cation, which lies on a twofold rotation axis, are Cl—Cu—Cl = 142.84 (4)°, Cl—Cu—S = 94.80 (2) and 99.97 (2)°, and S—Cu—S = 132.46 (4)°. The planes of the two imidazolium rings make a dihedral angle of 76.92 (8)°.

## Related literature

For structures of related compounds, see: Griffith *et al.* (1978 ▶); Kuhn *et al.* (1996 ▶).
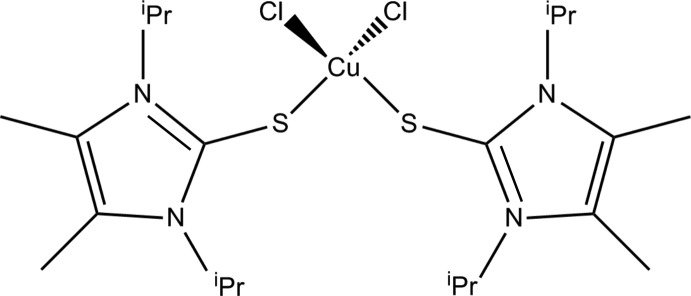


## Experimental

### 

#### Crystal data

[CuCl_2_(C_11_H_20_N_2_S)_2_]
*M* *_r_* = 559.14
Orthorhombic, 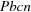

*a* = 14.0663 (12) Å
*b* = 13.1359 (11) Å
*c* = 14.9278 (13) Å
*V* = 2758.3 (4) Å^3^
*Z* = 4
Mo *K*α radiation
μ = 1.15 mm^−1^
*T* = 120 K
0.45 × 0.20 × 0.20 mm

#### Data collection

Bruker SMART CCD area-detector diffractometer
Absorption correction: multi-scan (*SADABS*; Sheldrick, 2004 ▶) *T*_min_ = 0.846, *T*_max_ = 0.991
26735 measured reflections
3418 independent reflections
2575 reflections with *I* > 2σ(*I*)
*R*_int_ = 0.055

#### Refinement

*R*[*F* ^2^ > 2σ(*F* ^2^)] = 0.043
*wR*(*F* ^2^) = 0.104
*S* = 1.07
3418 reflections
147 parameters
H-atom parameters constrained
Δρ_max_ = 0.52 e Å^−3^
Δρ_min_ = −0.32 e Å^−3^

### 

Data collection: *SMART* (Bruker, 2002 ▶); cell refinement: *SAINT* (Bruker, 2002 ▶); data reduction: *SAINT*; program(s) used to solve structure: *SHELXTL* (Sheldrick, 2008 ▶); program(s) used to refine structure: *SHELXTL*; molecular graphics: *SHELXTL*; software used to prepare material for publication: *SHELXTL* and local programs.

## Supplementary Material

Crystal structure: contains datablock(s) I, global. DOI: 10.1107/S1600536813006879/nc2307sup1.cif

Structure factors: contains datablock(s) I. DOI: 10.1107/S1600536813006879/nc2307Isup2.hkl

Additional supplementary materials:  crystallographic information; 3D view; checkCIF report

## Figures and Tables

**Fig. 1.**
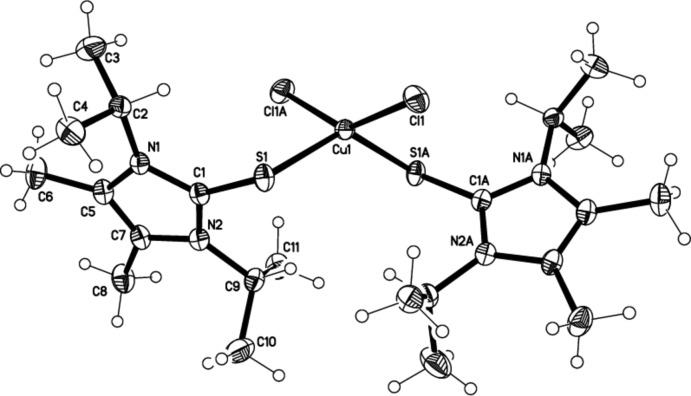
Molecular structure of the title compound with labeling. Displacement ellipsoids are drawn at the 50% probability level.

## supplementary crystallographic information

### Experimental

To a solution of 1,3-diisopropyl-4,5-dimethylimidazoline-2-thione (0.584 mg, 2.75 mmol) in acetonitrile (40 ml) CuCl_2_ H_2_O (0.168 mg, 1.25 mmol) was added and the mixture was stirred at room temperature for 48 h. Afterwards the solvent was removed under vacuum. Blue crystals were obtained from an acetonitrile solution by diethyl ether diffusion.

### Refinement

All Hydrogen atom positions were clearly derived from difference maps, then refined at calculated positions riding on the parent atoms with C—H 0.98 - 1.00 Å and isotropic displacement parameters *U*_iso_(H) = 1.2U_eq_(C) or 1.5U_eq_(CH_3_). All CH_3_ hydrogen atoms were allowed to rotate but not to tip.

### Crystal data

**Table:**

[CuCl_2_(C_11_H_20_N_2_S)_2_]	*F*(000) = 1180
*M**_r_* = 559.14	*D*_x_ = 1.346 Mg m^−^^3^
Orthorhombic, *P**b**c**n*	Mo *K*α radiation, λ = 0.71073 Å
Hall symbol: -P 2n 2ab	Cell parameters from 2598 reflections
*a* = 14.0663 (12) Å	θ = 2.5–22.4°
*b* = 13.1359 (11) Å	µ = 1.15 mm^−^^1^
*c* = 14.9278 (13) Å	*T* = 120 K
*V* = 2758.3 (4) Å^3^	Prism, blue
*Z* = 4	0.45 × 0.20 × 0.20 mm

### Data collection

**Table:**

Bruker SMART CCD area-detector diffractometer	3418 independent reflections
Radiation source: sealed tube	2575 reflections with *I* > 2σ(*I*)
Graphite monochromator	*R*_int_ = 0.055
φ and ω scans	θ_max_ = 28.2°, θ_min_ = 2.1°
Absorption correction: multi-scan (*SADABS*; Sheldrick, 2004)	*h* = −17→18
*T*_min_ = 0.846, *T*_max_ = 0.991	*k* = −17→17
26735 measured reflections	*l* = −19→19

### Refinement

**Table:**

Refinement on *F*^2^	Primary atom site location: structure-invariant direct methods
Least-squares matrix: full	Secondary atom site location: difference Fourier map
*R*[*F*^2^ > 2σ(*F*^2^)] = 0.043	Hydrogen site location: difference Fourier map
*wR*(*F*^2^) = 0.104	H-atom parameters constrained
*S* = 1.07	*w* = 1/[σ^2^(*F*_o_^2^) + (0.052*P*)^2^] where *P* = (*F*_o_^2^ + 2*F*_c_^2^)/3
3418 reflections	(Δ/σ)_max_ = 0.001
147 parameters	Δρ_max_ = 0.52 e Å^−^^3^
0 restraints	Δρ_min_ = −0.32 e Å^−^^3^

### Special details

**Table:**

Geometry. All e.s.d.'s (except the e.s.d. in the dihedral angle between two l.s. planes) are estimated using the full covariance matrix. The cell e.s.d.'s are taken into account individually in the estimation of e.s.d.'s in distances, angles and torsion angles; correlations between e.s.d.'s in cell parameters are only used when they are defined by crystal symmetry. An approximate (isotropic) treatment of cell e.s.d.'s is used for estimating e.s.d.'s involving l.s. planes.
Refinement. Refinement of *F*^2^ against ALL reflections. The weighted *R*-factor *wR* and goodness of fit *S* are based on *F*^2^, conventional *R*-factors *R* are based on *F*, with *F* set to zero for negative *F*^2^. The threshold expression of *F*^2^ > σ(*F*^2^) is used only for calculating *R*-factors(gt) *etc*. and is not relevant to the choice of reflections for refinement. *R*-factors based on *F*^2^ are statistically about twice as large as those based on *F*, and *R*- factors based on ALL data will be even larger.

### Fractional atomic coordinates and isotropic or equivalent isotropic displacement parameters (Å^2^)

**Table:**

	*x*	*y*	*z*	*U*_iso_*/*U*_eq_	
Cu1	0.5000	0.67838 (3)	0.7500	0.02220 (13)	
Cl1	0.63711 (4)	0.62457 (5)	0.80610 (4)	0.03363 (17)	
S1	0.56717 (4)	0.74956 (5)	0.62263 (4)	0.02594 (16)	
N1	0.44956 (13)	0.73957 (15)	0.47742 (12)	0.0214 (4)	
N2	0.41116 (13)	0.86096 (15)	0.56948 (12)	0.0232 (4)	
C1	0.47271 (16)	0.78364 (18)	0.55592 (15)	0.0218 (5)	
C2	0.50291 (16)	0.65185 (18)	0.44065 (16)	0.0254 (5)	
H2A	0.5421	0.6239	0.4908	0.030*	
C3	0.4384 (2)	0.5668 (2)	0.4097 (2)	0.0435 (7)	
H3A	0.3913	0.5522	0.4564	0.065*	
H3B	0.4763	0.5056	0.3980	0.065*	
H3C	0.4057	0.5875	0.3547	0.065*	
C4	0.57176 (19)	0.6862 (2)	0.36847 (18)	0.0361 (6)	
H4A	0.5367	0.7215	0.3210	0.054*	
H4B	0.6042	0.6267	0.3432	0.054*	
H4C	0.6188	0.7325	0.3946	0.054*	
C5	0.37268 (16)	0.7910 (2)	0.44012 (15)	0.0255 (5)	
C6	0.32782 (19)	0.7665 (2)	0.35256 (16)	0.0375 (7)	
H6A	0.2844	0.8215	0.3355	0.056*	
H6B	0.2922	0.7026	0.3577	0.056*	
H6C	0.3773	0.7592	0.3068	0.056*	
C7	0.34874 (16)	0.86563 (19)	0.49766 (16)	0.0261 (5)	
C8	0.27223 (19)	0.9433 (2)	0.48648 (18)	0.0379 (7)	
H8A	0.3010	1.0100	0.4752	0.057*	
H8B	0.2338	0.9464	0.5412	0.057*	
H8C	0.2317	0.9244	0.4357	0.057*	
C9	0.41332 (18)	0.92589 (19)	0.64987 (15)	0.0279 (5)	
H9A	0.4665	0.9003	0.6882	0.033*	
C10	0.4372 (3)	1.0351 (2)	0.6261 (2)	0.0581 (9)	
H10A	0.4944	1.0365	0.5887	0.087*	
H10B	0.4486	1.0739	0.6811	0.087*	
H10C	0.3840	1.0654	0.5932	0.087*	
C11	0.32354 (18)	0.9163 (2)	0.70488 (17)	0.0347 (6)	
H11A	0.2725	0.9555	0.6765	0.052*	
H11B	0.3350	0.9427	0.7653	0.052*	
H11C	0.3049	0.8445	0.7085	0.052*	

### Atomic displacement parameters (Å^2^)

**Table:**

	*U*^11^	*U*^22^	*U*^33^	*U*^12^	*U*^13^	*U*^23^
Cu1	0.0219 (2)	0.0220 (2)	0.0227 (2)	0.000	−0.00117 (16)	0.000
Cl1	0.0280 (3)	0.0363 (4)	0.0366 (3)	0.0100 (3)	−0.0029 (3)	0.0046 (3)
S1	0.0180 (3)	0.0380 (4)	0.0218 (3)	0.0020 (3)	−0.0010 (2)	0.0018 (3)
N1	0.0177 (10)	0.0267 (11)	0.0198 (9)	−0.0028 (8)	0.0009 (7)	0.0018 (8)
N2	0.0184 (10)	0.0283 (11)	0.0229 (9)	−0.0004 (8)	0.0016 (8)	0.0015 (8)
C1	0.0173 (11)	0.0272 (12)	0.0208 (11)	−0.0027 (10)	0.0033 (9)	0.0035 (9)
C2	0.0279 (13)	0.0237 (12)	0.0246 (11)	0.0027 (10)	0.0000 (10)	0.0004 (9)
C3	0.0509 (19)	0.0306 (16)	0.0488 (17)	−0.0065 (13)	−0.0044 (14)	−0.0060 (13)
C4	0.0337 (15)	0.0368 (16)	0.0378 (15)	0.0084 (12)	0.0102 (12)	0.0047 (12)
C5	0.0174 (12)	0.0355 (14)	0.0236 (11)	−0.0019 (10)	−0.0001 (9)	0.0023 (10)
C6	0.0285 (14)	0.0579 (19)	0.0263 (12)	0.0046 (13)	−0.0043 (11)	−0.0051 (12)
C7	0.0197 (12)	0.0344 (15)	0.0242 (11)	0.0005 (10)	−0.0003 (9)	0.0046 (10)
C8	0.0311 (15)	0.0493 (18)	0.0332 (13)	0.0120 (13)	−0.0022 (12)	0.0034 (12)
C9	0.0283 (13)	0.0322 (14)	0.0232 (11)	0.0021 (11)	−0.0006 (10)	−0.0040 (10)
C10	0.088 (3)	0.0421 (19)	0.0439 (17)	−0.0260 (18)	0.0083 (18)	−0.0099 (15)
C11	0.0338 (15)	0.0402 (16)	0.0301 (13)	0.0084 (13)	0.0076 (11)	−0.0034 (11)

### Geometric parameters (Å, º)

**Table:**

Cu1—Cl1^i^	2.2182 (6)	C4—H4C	0.9800
Cu1—Cl1	2.2182 (6)	C5—C7	1.346 (3)
Cu1—S1	2.3199 (6)	C5—C6	1.487 (3)
Cu1—S1^i^	2.3199 (6)	C6—H6A	0.9800
S1—C1	1.720 (2)	C6—H6B	0.9800
N1—C1	1.347 (3)	C6—H6C	0.9800
N1—C5	1.392 (3)	C7—C8	1.492 (3)
N1—C2	1.481 (3)	C8—H8A	0.9800
N2—C1	1.350 (3)	C8—H8B	0.9800
N2—C7	1.387 (3)	C8—H8C	0.9800
N2—C9	1.472 (3)	C9—C11	1.512 (3)
C2—C3	1.512 (3)	C9—C10	1.515 (4)
C2—C4	1.517 (3)	C9—H9A	1.0000
C2—H2A	1.0000	C10—H10A	0.9800
C3—H3A	0.9800	C10—H10B	0.9800
C3—H3B	0.9800	C10—H10C	0.9800
C3—H3C	0.9800	C11—H11A	0.9800
C4—H4A	0.9800	C11—H11B	0.9800
C4—H4B	0.9800	C11—H11C	0.9800
			
Cl1^i^—Cu1—Cl1	142.84 (4)	C7—C5—C6	127.8 (2)
Cl1^i^—Cu1—S1	99.97 (2)	N1—C5—C6	125.2 (2)
Cl1—Cu1—S1	94.80 (2)	C5—C6—H6A	109.5
Cl1^i^—Cu1—S1^i^	94.80 (2)	C5—C6—H6B	109.5
Cl1—Cu1—S1^i^	99.97 (2)	H6A—C6—H6B	109.5
S1—Cu1—S1^i^	132.46 (4)	C5—C6—H6C	109.5
C1—S1—Cu1	105.36 (8)	H6A—C6—H6C	109.5
C1—N1—C5	109.10 (19)	H6B—C6—H6C	109.5
C1—N1—C2	122.31 (19)	C5—C7—N2	107.6 (2)
C5—N1—C2	128.57 (19)	C5—C7—C8	127.4 (2)
C1—N2—C7	108.9 (2)	N2—C7—C8	125.0 (2)
C1—N2—C9	123.01 (19)	C7—C8—H8A	109.5
C7—N2—C9	128.1 (2)	C7—C8—H8B	109.5
N1—C1—N2	107.4 (2)	H8A—C8—H8B	109.5
N1—C1—S1	125.36 (18)	C7—C8—H8C	109.5
N2—C1—S1	127.19 (18)	H8A—C8—H8C	109.5
N1—C2—C3	112.6 (2)	H8B—C8—H8C	109.5
N1—C2—C4	110.8 (2)	N2—C9—C11	112.2 (2)
C3—C2—C4	112.7 (2)	N2—C9—C10	111.2 (2)
N1—C2—H2A	106.8	C11—C9—C10	113.0 (2)
C3—C2—H2A	106.8	N2—C9—H9A	106.6
C4—C2—H2A	106.8	C11—C9—H9A	106.6
C2—C3—H3A	109.5	C10—C9—H9A	106.6
C2—C3—H3B	109.5	C9—C10—H10A	109.5
H3A—C3—H3B	109.5	C9—C10—H10B	109.5
C2—C3—H3C	109.5	H10A—C10—H10B	109.5
H3A—C3—H3C	109.5	C9—C10—H10C	109.5
H3B—C3—H3C	109.5	H10A—C10—H10C	109.5
C2—C4—H4A	109.5	H10B—C10—H10C	109.5
C2—C4—H4B	109.5	C9—C11—H11A	109.5
H4A—C4—H4B	109.5	C9—C11—H11B	109.5
C2—C4—H4C	109.5	H11A—C11—H11B	109.5
H4A—C4—H4C	109.5	C9—C11—H11C	109.5
H4B—C4—H4C	109.5	H11A—C11—H11C	109.5
C7—C5—N1	107.0 (2)	H11B—C11—H11C	109.5
			
Cl1^i^—Cu1—S1—C1	26.05 (9)	C1—N1—C5—C7	−1.0 (3)
Cl1—Cu1—S1—C1	171.81 (9)	C2—N1—C5—C7	−179.6 (2)
S1^i^—Cu1—S1—C1	−79.98 (9)	C1—N1—C5—C6	178.6 (2)
C5—N1—C1—N2	0.8 (2)	C2—N1—C5—C6	0.0 (4)
C2—N1—C1—N2	179.55 (19)	N1—C5—C7—N2	0.7 (3)
C5—N1—C1—S1	−176.49 (17)	C6—C5—C7—N2	−178.8 (2)
C2—N1—C1—S1	2.2 (3)	N1—C5—C7—C8	178.2 (2)
C7—N2—C1—N1	−0.4 (3)	C6—C5—C7—C8	−1.3 (4)
C9—N2—C1—N1	178.81 (19)	C1—N2—C7—C5	−0.2 (3)
C7—N2—C1—S1	176.86 (17)	C9—N2—C7—C5	−179.4 (2)
C9—N2—C1—S1	−3.9 (3)	C1—N2—C7—C8	−177.8 (2)
Cu1—S1—C1—N1	−111.48 (19)	C9—N2—C7—C8	3.0 (4)
Cu1—S1—C1—N2	71.7 (2)	C1—N2—C9—C11	−116.7 (2)
C1—N1—C2—C3	132.1 (2)	C7—N2—C9—C11	62.3 (3)
C5—N1—C2—C3	−49.4 (3)	C1—N2—C9—C10	115.6 (3)
C1—N1—C2—C4	−100.6 (2)	C7—N2—C9—C10	−65.3 (3)
C5—N1—C2—C4	77.8 (3)		

Symmetry code: (i) −*x*+1, *y*, −*z*+3/2.

## References

[bb1] Bruker (2002). *SMART* and *SAINT* Bruker AXS Inc., Madison, Wisconsin, USA.

[bb2] Griffith, E. A. H., Spofford, W. A. III & Amma, E. L. (1978). *Inorg. Chem.* **17**, 1913–1917.

[bb3] Kuhn, N., Fawzi, R., Kratz, T., Steimann, M. & Henkel, G. (1996). *Phosphorus Sulfur Silicon*, **108**, 107–119.

[bb4] Sheldrick, G. M. (2004). *SADABS* University of Göttingen, Germany.

[bb5] Sheldrick, G. M. (2008). *Acta Cryst.* A**64**, 112–122. 10.1107/S0108767307043930 18156677

